# Parental perceptions of onsite hospital food outlets in a large hospital in the North East of England: A qualitative interview study

**DOI:** 10.1371/journal.pone.0205416

**Published:** 2018-11-02

**Authors:** Lorraine McSweeney, Suzanne Spence, Julie Anderson, Wendy Wrieden, Catherine Haighton

**Affiliations:** 1 The Human Nutrition Research Centre, William Leech Building, Newcastle University, Framlington Place, Newcastle upon Tyne, United Kingdom; 2 Child Health Research Strategy, Children’s Services Clinical Research Centre, Royal Victoria Infirmary, Newcastle upon Tyne, United Kingdom; 3 Social Work, Education & Community Wellbeing, Coach Lane Campus West, Northumbria University, Newcastle upon Tyne, United Kingdom; Northwestern University, UNITED STATES

## Abstract

**Background:**

Addressing the increasing obesity rates in children living in the United Kingdom has become a priority. A public health level approach as opposed to an individual approach is potentially one way forward. The wider food environment should be designed so that the ‘healthier choice’ is the easiest choice; this includes public sector settings such as hospitals. Many hospital outlets sell and promote food and drinks high in sugar, fat and salt undermining health messages developed by the UK National Health Service. Financial incentives have been provided to encourage hospitals to promote healthier food choices; however, few outlets have complied with the targets.

The aim of this qualitative interview study was to determine the dietary perceptions and needs of parents whose children attend a large children’s hospital in the North East of England and to identify potential barriers and facilitators to eating healthily in a hospital setting.

**Methods:**

Eighteen parents whose children attended the hospital as an in- or out-patient were recruited through either ward research nurses, information posters or a parent hospital Facebook page to participate in a one-to-one in-depth interview.

**Results:**

Parents reported a lack of affordable healthy options for sale both for themselves and visiting children. Although parents wanted to see more healthy options available for sale they did not feel it was appropriate to ban or restrict sales of any food types. Parents of frequent or long-term in-patients found it difficult to adequately feed themselves.

**Conclusions:**

The ways in which visitors and staff can be encouraged to choose the healthier option in an NHS hospital setting warrants further investigation. The use of ‘nudge theory’, which has gained particular momentum in areas such as health promotion, may be a tool which can be utilised by hospitals to facilitate the promotion of healthy eating.

## Introduction

As reported by the UK National Child Measurement programme, a third of 10–11 year olds and over a fifth of 4–5 year olds are overweight or obese [1]. Being overweight or obese increases the risk of myriad health morbidities costing the National Health Service (NHS) more than £5 billion every year [2]. Hospitals have a role in addressing the increasing obesity burden by helping visitors choose a healthier lifestyle [2]. It is important that health professionals healthy eating advice, routinely given to their patients, is not undermined by a lack of healthy food and drink items offered for sale on hospital premises [3]. However, despite NHS trusts having contracted food standards for feeding patients [4], there is no regulation for the wider hospital food environment. Onsite outlets sell and promote foods high in salt, sugar and fat [5]. Private Finance Initiative deals mean that many hospitals do not own their own premises, but lease them from buildings consortia, who also rent out areas to commercial operators [6]. Economic reliance on revenue may be a key motivating factor encouraging the growth of outlets selling less nutritious food in hospitals [7].

The ‘food environment’ where people work and live is likely to influence what people eat [8] with unhealthy food environments fostering unhealthy diets [9,10]. Evidence of the impact of the hospital food environment in the UK is limited. The UK Government’s recent childhood obesity strategy [11] stresses the importance of every public sector setting, including hospitals, having a food environment so designed that the easy choices are also the healthy choices. Hospitals represent an environment which has great potential for influencing what people eat; marketing practices, such as pricing, food placement and signage can change the way visitors eat [8,12]. Furthermore, there has been a shift in how the ‘obesity epidemic’ should be tackled; the focus for action is moving towards a public health level, which creates supportive environments [10] as opposed to an individual level approach.

In 2016, after pressure and support from the Campaign for Better Hospital Food [13], NHS England offered financial incentives for hospitals to remove price discounts and advertising for junk food and sugary drinks and to provide healthier alternatives [14]. The targets are known as the Health and Wellbeing Commissioning for Quality and Innovation (CQUIN) [15]. Compliance with the targets was mixed with several well-known retailers only fulfilling one of four specific targets such as ‘ban price promotions on sugary drinks and foods high in fat, salt and sugar’ [16]. As expressed by Malhotra (5), an interventionist cardiology specialist registrar, “the obesity epidemic represents a public health crisis, but it is a public health scandal that by legitimising junk food hospitals have themselves become a risk factor for diet related disease”.

## Methods

### Aim and design

The aim of this qualitative semi-structured, in-depth interview study was to explore the perceived needs of families regarding the types of food that are offered for sale in shops and outlets in a North East of England hospital and the potential barriers and facilitators to accessing healthy food options.

### Setting

A large children’s hospital in the North East of England. The hospital is one of the four largest major children’s hospitals in the UK, approximately 73,000 children engage with the service each year.

### Participants, recruitment and consent

Participants comprised a purposive sample of parents of children (age 0–16 years) attending the children’s hospital either as an in-patient or out-patient. This was to enable a representation from the diverse population which attends the hospital. The hospital Children’s Services Research Unit contributed in the identification of potential participants; this was achieved through the use of ward research nurses and trained research champions (research champions support research activities within their department and signpost patients and staff to research advice or resources). Parents also had the opportunity to contact the study researchers directly by email or telephone as study information was advertised via study posters and information sheets placed in out- and in-patient departments and in on-ward parent rooms/kitchens. In addition, a poster advert was placed on the hospital parent group Facebook page.

Interested parents were provided with a study information sheet, if happy to be contacted by lead researcher (LM), they were asked to complete an Expression of Interest form (EOI) which captures the participant contact details. LM contacted the parent to arrange a convenient time and location for the interview. Written informed consent was obtained at the beginning of the interview. Patients received a shopping voucher to thank them for their participation.

Face-to-face interviews were conducted by LM and lasted between 15–30 minutes. Interviews were conducted until data saturation was reached, whereby no new themes were identified.

### Ethics

Ethical approval was granted by the Health Research Authority, Rec reference: 17/LO0520. Participants were informed that they were free to leave the study at any point without having to give a reason.

#### Materials

The interview topic guide was developed from a review of the literature and in consultation with the Newcastle University Teaching Hospitals Healthy Eating Policy committee. The guide was reviewed by a local Young Person’s Advisory Group (YPAG North East) and a local virtual advisory parent group.

The topic areas included: experience of being a hospital visitor; knowledge and use of hospital food outlets; satisfaction of available foods; perception of regulations; and on-ward catering. The topic guide is available on request from the corresponding author.

#### Data management and statistical analysis

Interviews were digitally recorded with the participant’s consent and transcribed verbatim. Framework analysis was adopted which allows the reduction of data through summarisation and synthesis enabling comprehensive and transparent analysis [17]; its use is increasingly common in healthcare research [18]. NVivo software was used to aid indexing and charting [19]. Guided by the principles of grounded theory [20], the data was repeatedly read and coded independently by LM within a framework of a priori issues identified from the topic guide and by participants or which emerged from the data. Data reduction and coding was a continuous process; this aided the decision for data saturation and when to conclude recruitment and interviews. Regular discussion and review of the analysis by LM and the project team acted as a quality control measure.

## Results

Five parents expressed an interest to take part in the study having been approached by ward research staff or by picking up an information leaflet on the ward. The Facebook advert yielded 21 interested parents. In total the final number who consented to an interview was 18 parents; the remaining parents did not respond to follow-up contact made to organise an interview. Thirteen of the parents were mothers (Table 1) and the majority of the children were in-patients of which six children were classed as long-term patients. Three parents did not disclose their child’s visit status. Thirteen parents were interviewed at the hospital either, in their child’s room, in the ward parent room, or on the food outlet concourse. Other parents were interviewed in their own home or workplace.

**Table 1:** Participant characteristics.

Gender of parent	Male	Female				Total
	5	13				18
Status of child	Out-patient	In-patient (short term)	In-patient (long term)	Both	Unknown	
	1	7	6	1	3	18
Interview location	Hospital	Parent’s Home	Parent’s Workplace			
	13	4	1			18

The findings are presented by the following themes which emerged from the data: Parent’s purchases are influenced by cost and speed; hospital outlet food choice is lacking but should not be restricted; quality of hospital outlet food is variable and eating environment poor; maintaining a healthy diet is difficult; some but not all wards have self-catering facilities for parents; and, some but not all wards provide parents with food. The coding framework matrix can be seen in S1 Appendix. The themes which emerged from the data as recorded by parent/patient type are displayed in Table 2.

**Table 2:** Themes identified by type of parent/patient.

Identified theme	No of parents contributing to theme (Total n = 18)	Type of patient				
		In–patient	Long Term	Out—patient	In- and out—patient	Not known
Parent’s purchases are influenced by cost and speed	14	6	3	1	3	1
Hospital outlet food choice is lacking but should not be restricted	13	4	5	1	0	3
Quality of hospital outlet food is variable and eating environment poor	10	3	3	1	0	3
Maintaining a healthy diet is difficult	7	2	3	0	0	2
Some but not all have self-catering facilities for parents	11	3	6	0	0	2
Some but not all wards provide parents with food	6	2	2	0	0	2
Parents were unaware that the NHS lacked control over hospital food outlets	9	2	3	1	0	3

Each theme is illustrated by examples of parent quotations. Quote references: parent/ID number plus whether child is an in-patient (IN) or out-patient (OP).

### Parent’s purchases are influenced by cost and speed

Cost was reported as a major determinant of the types of purchases made by parents in the food outlets. This was especially so for parents of children who spent long periods of time in hospital:

*“Yes, I know there's a few options, but I will be honest and say I have avoided quite a bit because of the cost. I just think for the extra walk to the other shops [located out-with the hospital], I think it's… I mean, for example, we went for a bottle of water when we were first here, and I think it was like £1.75 for a bottle of water and I just thought it's just daylight robbery, really*.*” (P25IN)*

Some parents felt that visitors were being targeted as a ‘captive audience’:

*“I know a lot of places do it [increase costs], but it is not right just to hike the prices because you have a captive audience and that sort of there. It is like when you go on holiday in the caravan parks, you go to their shop, and it is massively more expensive. It is slightly different if you are on holiday you are choosing to be there, but in a hospital, you do not often get the time to plan. You find yourself there in quite upsetting circumstances so when you are going into the shops and you are paying three times more than what you would if you had the time to go to [name of UK supermarket] on your way in from … on your way there*.*” (P25IN)*

After cost, some parents tried to think about the healthiness of the food they were choosing:

*“Well cost is a thing and then I’d like something that’s fresh, so I look at the freshness of it and try to pick something quite healthy*.*” (P20IN)*

Speed of purchase was important for some. Parents who had a child on a ward were very reluctant to leave them for any amount of time, therefore, they were looking for something that could be purchased in the least amount of time. Inevitably, outlets with longer queues were often avoided:

*“Within the canteen [hot food outlet] itself, because normally if we’re coming at lunchtime, it’s so busy and if my little boy is ill, if somebody comes in to watch him, I still like… to leave him for a long period of time and you could be waiting, like 20, 30 minutes in the queue. So, quite often it’s just grabbing a sandwich and then running back to the ward with it*.*” (P18IN+OP)*

However, parents were not always in the right frame of mind to think too much about the type of food to purchase:

*“Most of the time; it is … you know you are in survival mode when you are in hospital with your child, so you are just looking to grab something quick which will fill you up*.*” (P017IN)*

#### Hospital outlet food choice is lacking but should not be restricted

There was an overarching view that variety of food types was lacking, especially for children, vegetarian and healthier options:

*“I think they try to because they do have some children's meal options but it's really unhealthy children's meals options, like, you can tell the fish fingers, the chicken nuggets are deep fried…It's a hospital and they're deep frying food for children. It's not good, but then, when I bring my daughter with us, she's four and a half, she literally, has to have fish fingers and chips because there's no other options for her and I know that that's not a healthy meal for her*.*” (P19IN)*

Parents identified ‘healthier’ choices as being salads, fruit, fresh vegetables, jacket potatoes, breadsticks, rice cakes and cous cous. Options considered ‘unhealthier’ included: bacon sandwiches, sausage and chips, fizzy drinks, chocolate and sweets, gingerbread men, crisps, pizza, chips, and fish fingers and chicken nuggets that had been deep-fried. Despite parents reporting a lack of, and stating a desire for, more healthy options, it was apparent that a ban of, or a restriction of certain food types would not be a popular move:

*“Personally, I think I would have hated that [a ban on certain foods]. I think they've got a place, and I think especially as an adult I think you have to be able to make your own informed choice. I just think it's got to be balanced. It's being able to have, you know, a bar of chocolate if you want a bar of chocolate but it's also being able to have some apples and oranges*.*” (P06OP)*

Moreover, it was widely accepted that food was more than something just to satisfy hunger. It was acknowledged that in times of stress, food could be used for comfort or to soothe a child who had to go through a medical procedure:

*“I suspect that’d be a lot of people would be up in arms…if I’m honest and that includes the parents and the grandparents because I think a lot of people do feel sorry for the child who is in hospital and they would treat all day, you are having blood test, you are having this done and people treat food and this is huge emotional thing that goes with food as well as the nutritional side of thing*.*” (P08OP)*

Furthermore, one parent reported that children who had lost their appetite were encouraged to eat anything:

*“We were on the kid’s ward so you can't… When the children, especially on the cancer ward, when they're on steroids and things, you kind of… if they stop eating, you’re literally feeding them what they want*.*” (P19IN)*

#### Quality of hospital outlet food is variable and eating environment poor

Parents reported issues with freshness and ways in which food was cooked and presented in the hot food outlet:

*“It is horrendous, it is really bad. It is very expensive, and the food is, for example, I got a jacket potato and the skin was hardly baked. I could not even cut it. It is food that they have obviously cooked in mass and is kept warm, but what happens then … It is the same with the sausages … they are so … I remember getting sausage, chips and beans and it was pretty much inedible. It was really bad. Especially for what you are paying, you would not mind paying the extra, it’s as if they just think ‘right, they are stuck here, these people are stuck here, they have nowhere else to go, they will pay whatever,’ and it is grim if you want a meal*.*” (P05IN)*

It was conceded that items purchased from well-known branded outlets such as Costa and Subway were of better and familiar quality:

*“Costa as well. I find that is the most … it is expensive, but you get the … the food you get there is better quality than upstairs [where the hot food outlet is located], so I tend to, if I’m wanting food, go to Costa*.*” (P19IN)*

Linked to quality of purchases was satisfaction with the food concourse environment. Some parents felt the area was too open and noisy and needed to be more child-friendly:

*“It's very noisy and open environment and it's all hustle and bustle and I think when you're stressed, and your little girl is in hospital, sometimes you just want to go to a bit more of a softer place*.*” (P010IN)*

The positioning of snacks and sweets in the shop was a concern for some:

*“Going in the shop downstairs with my daughter, it's, like, as soon as you go in, it's sweets and chocolate and gingerbread men and things like that, and then there's a tiny little section of fruit. So she doesn't see that fruit, she's, like, “Can I have some chocolate, can I have some gingerbread men?” And you're, kind of, in this kind of environment, you want to do whatever you can to avoid the tantrums so you cave and you give it, but, if it wasn't there in your face, and there was, actually*, *more healthier appearing choices for children” (P19IN)*

#### Maintaining a healthy diet is difficult

It was apparent that maintaining a healthy diet whilst being a visitor to the hospital was considered a difficult task. This was especially so for parents of children who had to stay in hospital for long periods of time:

*“I kind of think certainly it never seems to be particularly healthy, you know? I think things like fresh vegetables and salads and things tend to be, you know, few and far between, and I think especially when you're in for a long time*.*” (P19IN)*

It was felt that the healthy eating messages on posters being promoted in the outlet seating areas did not correspond with the types of foods being sold:

*“I've noticed in the outlet there's always these pull-up stands that have like, I forget what's on them now, I've looked at them 100 times as well, about red meat and different things on the pull-up, and I've noticed those, but they never seem to correlate with what they serve, you know? They have all these healthy pull-up stands and I think yeah, but you're serving chips and sausages and curry, and, you know, when you just want to be able to try to have something a bit healthier*.*” (P10IN)*

Even though the majority of parents were opposed to unhealthy food options being removed from the hospital outlets, they described several ways in which they would like to see outlets promoting healthier eating:

*“Just by changing the menus a bit, changing what they're offering, even just as simple as just offering something like a bit like what Subway do but in the other café bit so like salad bowls, jacket potatoes*, *that sort of thing that aren't fried or that aren't covered in oil or… you know?” (P18IN)*

*“Perhaps making their food more affordable. You could incentivise it, if you are buying a healthier option then it is cheaper than buying a portion of gravy and chips, which would normally be the cheapest thing on the menu and therefore, why people would head toward that option*.*” (P19IN)*

Promoting healthy options in the right way for children was considered important for some:

*“I think it's all about what's presented to them as well. I think. I mean there's lots of really cheap and cheerful places that seem to do okay. And even like I think [name of UK supermarket] cafés and stuff where they'll do, you know, sandwich deals and things where you can get a fruit bag instead of a bag of crisps…or you can do a little kid's box that's got sultanas and stuff in instead of crisps or… I think there's lots of… it doesn't have to be reinventing the wheel, I think. I think it's just about having…having choice, and having it in a good place*.*” (P25IN)*

*“Another thing, I mean, which [name of UK supermarket] do, and I know it's a long shot, but they give free fruit to children… I think it's a brilliant encouragement. You know, maybe you could, if you look at things like that*.*” (P19IN)*

#### Some but not all have self-catering facilities for parents

Some wards had facilities that parents could use like kettles, fridges and microwaves, although it was evident from the parent responses that these facilities differed greatly between wards. However, despite some parents having access, this too presented difficulties as parents still had to think about buying or bringing in food items:

*“Some wards are really very good for parents and some aren't. I mean some wards have kitchens with microwaves and fridges and all sorts, but the ward where my little girl went after intensive care I think had been a converted day ward and it was, it had nothing for parents. They had a kitchen that they used to use to serve their meals, but it was locked. So if I wanted a glass of water, I mean I could risk the water at the tap which I wasn't really very happy doing, or I could just stock… I was just stockpiling bottles of water. It was like being in quarantine*.*” (P10IN)*

Other difficulties for parents included a lack of storage space for fresh foods, foods needing to be labelled and food going missing:

*“Yes, yes, as long as you label it; otherwise it gets pinched. [Laughter] People do do that so yes. Yes, I didn't know that at all. I was just surprised*.*” (P10IN)*

However, although parents were frustrated with the on-ward food facilities for themselves, they had nothing but praise about the food provided to their children:

*“The staff are so good and the food that she’s fed, and they are there with the drinks refreshed and … It is just she is well looked after*.*” (P20IN)*

#### Some but not all wards provide parents with food

Some parents spoke of being provided with food when on wards with their children. As with the cooking facilities, this service appeared to differ greatly between wards. Some parents had been provided with hot drinks or a snack box. On some wards parents could order and purchase meals from the children’s ward menu:

*“So, some wards, not all of them, it’s normally like Ward 6 which gets emergencies coming in, they have a snack fridge, it’s meant to be for the children, the patients where they have a few little sandwiches, some yoghurts, some cheese and biscuits and that kind of thing in there. Sometimes if I’m on a ward where they have that facility, the nurses will bring me a sandwich*.*” (P20IN)*

Breast-feeding mothers were automatically given food and another mother (non-breastfeeding) was told she could claim some tinned produce but this was not felt to be acceptable to her.

Parents described how a service where parents could pre-order food via an app, to avoid outlet queues, or have sandwiches/food delivered directly to the ward would be invaluable:

“*What I would like*, *in an ideal world*, *is for the parents that are in hospital on a regular basis*, *if there was somewhere where we could ring and order food and get it delivered to the ward or have a time when we’re free to go and pick it up*.*” (P19IN)*

It was also strongly felt that long-stay parents should be issued with a discount card scheme to help with the ongoing costs. Especially as a discount service was available in other hospitals.

#### Parents were unaware that the NHS lacked control over hospital food outlets

Many parents were surprised to find out that the NHS did not have any control over the types of outlets and foods sold on hospital premises. It was felt that the types of foods being sold contradicted health messages:

*“I mean I know they're not NHS outlets but I'm surprised that they haven't got some kind of governance there because it just seems… I mean it's almost tantamount to being able to smoke in a hospital, isn't it? I mean they've put that message out that not being able to smoke on NHS property. But then you can eat fatty food and have all these kind of unhealthy things and it kind of goes against the principle*, *doesn’t it?” (P19IN)*

*“I think the NHS should definitely have a say in what is going on, it is in their hospital and affecting their patients and their quality of stay. I will be honest it is the only negative thing I have to say about the [name of hospital]*.*” (P25IN)*

There was a belief that well known branded outlets who would not necessarily be regulated by the NHS, should at least have to follow NHS healthy eating guidelines.

## Discussion

The aim of this study focused on ways in which visitors to public buildings such as hospitals could be encouraged to choose healthier options when purchasing food items. By interviewing a targeted population of visitors (parents) attending a busy children’s hospital it was hoped that barriers to achieving this goal and facilitators to implement change for all types of visitors could be identified.

We identified several barriers facing visitors such as cost; availability and variety of foods; environmental factors such as length of queues, ambiance of surroundings; and, social and emotional aspects such as using food for comfort or eating anything just for convenience and satiation. Parents reported a desire for healthier options for themselves and their children to be available. This and other recommended facilitators are reported in the ‘Implications for policy’ section below. There was surprise that food sold in NHS outlets was not regulated, some parents even noted the apparent disconnect between health messages being promoted and the types of foods being sold. However, there was very strong opposition for the banning or restriction of certain types of foods, namely foods/snacks high in sugar, salt and saturated fats. It was felt that people should have the right to choose and government bodies should not dictate what people should and should not eat.

Research exploring the wider hospital food environment in the UK is scarce. In the US and Canada several studies have been conducted to implement interventions with the aim of encouraging healthful eating [8,21–25]. Some studies have focused on changing the vending machine environment [26–28] for example by stocking ‘better’ or ‘other’ choices. In New Zealand the District Health Boards are in line to adopt a new Healthy Food policy across the country. This policy provides guidance on the types of food suitable for sale in hospitals; foods categorised as ‘red’ (foods high in salt, sat fat and sugar) will not be permitted for sale. It has been suggested that such interventions may have other positive effects such as modelling healthier choices, providing supportive environments and influencing social norms [28].

In concordance with the findings from this study, a recent Australian study found that 90% of surveyed parents felt that hospital outlets should sell mostly healthy food. However, in contrast with the present study, 83% of the Australian parents felt the health service should restrict the sale of unhealthy food and drink [3]. Moreover, it has been suggested that fast food outlets on hospital premises are perceived to be familiar and reliable [29], which appeared to be the case with the well-known branded available outlets. However, this may simply be because the alternatives were not satisfactory.

Some parents in this study felt that occasionally eating the types of food offered for sale at the outlets would not be harmful in the short-term. However, a profound unexpected finding is the impact the food environment is having on parents of frequent and/or long stay patients with respects to both their dietary health and financially. Many parents were existing on snacks and sandwiches, with some not having anything to eat all day or being able to access basics like drinking water.

The ways in which visitors and staff can be encouraged to choose the healthier option in a hospital setting warrants further investigation. The use of ‘nudge theory’, which has gained particular momentum in areas such as health promotion [30], may be a tool which can be utilised by hospitals to facilitate the promotion of healthy eating to visitors in a non-paternalistic way. A nudge is described as ‘an aspect of the choice architecture that alters people’s behaviour in a predictable way without forbidding any option or significantly changing their economic incentives’ [31] (p.6). A nudge can involve making an environment less conducive to someone making an unhealthy choice; provision of information; changes to default; and the use of norms [32]. Most eating behaviour occurs without much conscious effort [33] and people’s behaviour is susceptible to the influence of ‘default rules, framing effects and starting points’ [34]. It could be argued that re-framing the environment to change people’s behaviour and to ‘stop them making the ‘wrong’ decisions’ is controlling and goes against public wishes. However, private industry and corporate actors have relatively free rein in influencing their customer’s health behaviours [34]. This reiterates the unease of retail franchises being present in hospitals.

### Implications for policy

Several recommendations which may improve visitor’s experiences of the hospital food environment have emerged from the study findings:

1. Ensure outlets provide healthier options such as baked potatoes, salads etc. at a reasonable cost (i.e. in line with shops out-with the hospital).
2. Provide healthier options for children and vegetarians.
3. Cook meals in smaller batches to safeguard freshness and satisfaction of taste.
4. Provide long-term visitors with a loyalty/price reduction card/scheme.
5. Set up a pre-order/delivery service for parents confined to wards.
6. Standardise on-ward parent facilities.
7. Provide free drinking water.
8. Ensure outlets prioritise displays of healthy foods/snacks over unhealthier choices.

### Strengths and limitations

It is believed that this study investigating the types of food and drinks sold to visiting children and their families in the UK is the first of its kind. Our study was conducted in a single, large-scale hospital, thus generalisability to other healthcare settings may be limited. In addition, we recognise as is common in health care research, parents who volunteered to participate in the study may have prior health knowledge or interest in health-related matters; this may impact the findings. However, the in-depth nature of the interviews enabled a rich source of data to be collected.

## Conclusions

Foods currently being sold in many NHS outlets are undermining public health messages. The findings from this study highlight the difficulties hospital visitors may face in achieving a healthy diet. Study participants stated a desire for better food choices and voiced concern over lack of NHS legalisation. In the wider context, providing a better food environment may promote long-term, positive health behaviours. This in turn may contribute to changing hospital food policies benefiting not only visitors and their children but all patients and staff also.

## Supporting information

S1 Appendix Coding matrix table. (DOCX)
