# Supplementary material for: Parental perceptions of onsite hospital food outlets in a large hospital in the North East of England: A qualitative interview study
Source: PLoS One. 2018 Nov 2;13(11):e0205416. doi: 10.1371/journal.pone.0205416 (PMC6214497; doi:10.1371/journal.pone.0205416)
Supplement: S1 Appendix — (DOCX) [file pone.0205416.s001.docx]

| **Coding Matrix** | **Example coding themes** | **Concepts** |
| --- | --- | --- |
|  |  |  |
| **1 – On ward eating** | - Some wards have tea/coffee making facilities - Some wards have fridges and microwaves - Food in shared parent storage can go missing - Difficulties in obtaining basics such as drinking water - Finds it stressful/difficult to leave child - Long-term stays make it difficult to organise shopping/cooking/eating - Some wards provide snack boxes - Parents are ‘tied’ to the ward - Easier to rely on snacks - A sandwich pre-order/delivery service would be beneficial - Frequently buys food/snacks from the outlets to be eaten on the ward - Lone parents on wards often don’t/can’t eat all day - Food from the child’s meal service could be ordered but had to be paid for - Parents reliant on others bringing food to them - Was offered food on the ward but did not rate the quality - Mums get together to order a take-away delivery | - On ward facilities available to parents are mixed - Some wards will occasionally offer food to parent - Parents are often unable or do not want to leave their child on the ward - Parents tied to a ward find it difficult to obtain food and drink - Long-stay parents find it difficult to think about their own shopping, cooking and eating - Food eaten on ward is often bought from external sources - A ward delivery service would be of benefit |
| **2 – Purchase influencers** | - Children’s meals are too expensive - Cost and healthiness of food - Only buys meals onsite if essential - Feels it is better value to purchase healthy foods off-site - Tries to make hospital visits a pleasant experience for child by buying food treats - Food is being targeted as a luxury item which is wrong - Café health promotional posters do not correspond with the types of foods being sold - Finds it difficult to decipher what the hot meal options are - The length of queues and busyness of an outlet will determine a purchase - An upset child needs a treat - Costs for long-term patients needs to be assessed - Speed – how quickly a purchase can be achieved away from the ward - Costa is best for taste but options cannot be consumed everyday - Not every outlet is family friendly - Tends to use Subway as options are familiar - Prefers to bring in own food to minimise costs - Vegetarian choices are limited - Looks for food that is quick and filling - Tries to look for healthy options - Canteen needs to improve greatly to compete with chain outlets - Children are unable to choose a child portion of an adult meal so usually end up with chips - The outlets only cater for ‘normal’ families - Does not have any choice over what to purchase when tied to hospital - Finds it difficult to carry out healthy eating plan - Choices very limited - Costs determine choice - Bottled water is too expensive - Has seen offers on soft drinks - Sandwiches are expensive - Likes having the option of a Subway but wants more healthy choices | - Cost is a major determinant of onsite food purchases - It is better value to buy food externally - Visitors are being targeted as a captive audience - Food treats are purchased to calm anxious children - Choice and variety of options in café is limited - Speed of purchase and satiety of food choice can influence buying behaviour - Branded outlets are liked but not considered appropriate for every day consumption - Healthy option are limited - Children’s options expensive and limited |
| **3 – Purchase satisfaction** | - Would like more affordable options for children - Onsite food choice is lacking - Costa is no more expensive compared to off-site but M&S would present better value - Unhappy that hospitals are able to target visitors with high costs - Health promotion stands do not correlate with the types of food being sold - Finds identification of hot food difficult and costs are too high - There is too much unhealthy food on offer - Food choices are too greasy – too many fried foods on offer - Food is very expensive to buy - Costa purchases are tasty but not suitable for eating every day - The quality of the food often inedible and overpriced. - People have no choice in having to purchase food onsite - Prefers to purchase food externally due to high costs - Would like more option of healthier choices in the evenings - Café food does not look fresh - Water is very expensive - Subway staff are not aware of halal practices - Food ok for children but not the overall environment - Timing of food quality is erratic but always costly - Child always has to have fish fingers and chips – does not feel this is a healthy option - Child advertised promotions are not always given - Children do not like the available options - Food is filling but not healthy - Too much pre-packaged and not enough fresh food | - Child options are not highly rated and considered expensive - Dissatisfaction with visitors being targeted with high costs - Health promotion messages do not correlate with foods being sold - Too many fried and packaged foods on offer - Quality of food and availability of healthy options erratic - Some outlet staff unaware of Halal practices - Costa outlets are liked but choices not suitable for every day consumption |
| **4 – Perceptions of health** | - Healthy option meals limited especially for vegetarians - Eating take-away food every day has induced a 2 stone weight gain - To restrict some foods would be a good idea but food has emotional attachments, people would find it difficult - Getting healthy options as a long-term patient is difficult - Giving people more cheaper healthier options like fruit bags can be feasible - Junk food has its place and people should be able to choose but healthy options should be available also - Café should offer more healthy options - Foods should not be banned, most people are sensible and can make good choices - More salads and vegetables should be made available - Lack of access to fruit and vegetables for long-term visitors - The healthiest choices should be the cheapest ones - People should have choice if they want fizzy drinks - Smoking and drinking are not permitted promoting healthy living should be across the board - More choice should be available with small fresh batches cooked during the day - More fruit variety would counterbalance a lack of ‘Junk’ choice - Would support a junk ban as pestered by daughter for sweets and biscuits - People should be able to make their own choice regarding health - The price of healthy food should be improved - Outlets should provide information about their healthy eating options | - Difficult to purchase healthy options especially as a vegetarian or long-stay visitor - Patients and visitors should have access to all types of foods and be free to choose what to eat - More healthy, reasonably priced options should be made available - Outlets should provide information about their healthy eating options |
| **5 – Perceptions of choice** | - There is not a lot choice - Limited choice for dietary restrictions - Child options should be more affordable - Would like more salads with a range like M&S - There should be more family friendly options - Emotionally people need all types of food to choose from - People should be able to make an informed choice from a range of food - There are too many unhealthy food options - Junk food should not be banned – healthy options should be sold alongside - Is more likely to buy food externally - When tied to the hospital there is no option but to buy food onsite - Not enough vegetarian/halal choice - Items for sale need to be cheaper in comparison with supermarkets - More choice with discount options should be available - Child cannot have a smaller version of adult meal - Would like options that do not include mashed potato or chips - Labelling on foods/meals erratic and confusing - Children’s meal options are not healthy - Café food not nice – Subway choice is limited - Children not eating need to be able to choose any food items that they want - Specific foods should not be banned people will just buy elsewhere - Family options erratic - Would like more independent outlets with locally sourced fresh food - Banning perhaps too far but would support some restrictions | - Choice of healthy options is limited - Child and special dietary options are limited - Patients and visitors should have access to all types of foods and be free to choose what to eat - A wider range of fresh foods should be available |
| **6 – Perceptions of environment** | - Outlets are expensive – hospitals should be functional and not classed as a luxury good - The outlet environment is too noisy and too open – somewhere less open would be appreciated - Queues for Subway are too long it’s the only place to get something healthy - Environmental experiences on the ward are excellent from on-ward catering and cleanliness – the outlet environment lets the hospital down - Happy with outlet opening hours but not with the quality of the purchases - A hospital environment should not be using visitors as a captive audience - Outlet workers/servers are not always aware of halal practices - Frustrating that parents are not able to receive discount on food - The outlet eating area needs to be more child friendly in respect of space and activities - Too many unhealthy options in full view of children | - Dissatisfaction with hospital treating visitors as a captive audience and charging excessive prices - The outlet eating area could be more inviting and child-friendly - The outlet environment lets the hospital down |
| **7 – Practical considerations** | - Is unable to make toast for child due to health and safety - Found it difficult to obtain even a glass of water - Fridges and microwave are available on some wards but still reliant on someone bringing food to the ward/parent - Difficult to worry about own eating/health when tied to ward - Would like an app where you could pre-order food and collect without having to wait in a queue - Most wards have parent’s rooms with fridge but food needs to be labelled - When staying with child does not have time to go shopping - Can use facilities in parent room but food goes missing - Parent can only leave child for 5-10 minutes at most - When parents are tied to ward a sandwich delivery system would be beneficial - A fresh food delivery system would be beneficial - Reheating food in a microwave is not always satisfactory - Having to ‘scrub up’ again after leaving ward for a period of time is stressful - Not able to shop and cook for self when stuck on the ward - Most wards have a fridge but you are still reliant on others to bring food to you - Some wards have snack fridges for children and sometimes parents are offered a sandwich - If someone doesn’t visit during the day the parent often will not eat. A sandwich delivery service would be ideal - Unable to store fresh food on the ward and space is very limited | - Parents tied to a ward with child have difficulty in achieving a satisfactory diet - Parent on-ward facilities vary between wards - Parents can be reliant on others to provide them with food and water - An on-ward food pre-order/delivery service would be of benefit to parents |
| **8 – Services for parents** | - Mother was breastfeeding so was given food on the ward - Never heard of a food policy - Has been given a snack box at night - Was given snacks and hot drinks on A&E ward - Was offered tinned food only - Some wards will let parents eat any additional food from children’s meal service - Has been offered sandwiches and breakfast previously - Has asked for food as some days has gone without eating. - Has been given sandwiches from the snack fridge - Was never offered food - Had the option to order and pay for a meal from the child’s ward menu - Has been told that there is no food available for parents - A food compensation policy would help long-term families - A ward health care assistant has offered food to parents | - On-ward food provision varies between wards/departments - Parents are unaware of any hospital food policies - A food compensation scheme for parents of long-term patients would be of benefit |
| **9 – Views of onsite policies** | - How much money will the NHS be given to regulate food sales? - Hospitals have to generate an income - The NHS should be able to regulate food outlets as they regulate no-smoking policies - Surprise at NHS not regulating outlets - NHS should have authority over the outlets. - The food outlet provision is the only negative aspect of the hospital - Hospitals should not be run as a business - The café should improve its facilities and reduce costs – which would be better than the branded chain outlets - If the hospital is not benefitting from the outlets they should not be allowed - It’s wrong that private companies can profit from NHS patients - Expected the café to be an NHS regulated outlet – no strong feelings about it - Outlets should still have to follow healthy eating regulations | - NHS should regulate what outlets are allowed to sell in hospitals - Hospital food facilities should be improved without penalising visitors |
